# Case report: therapeutic monitoring of vancomycin in an acute liver failure patient with anuria under high-flow continuous hemodiafiltration

**DOI:** 10.1186/s40780-023-00283-0

**Published:** 2023-05-01

**Authors:** Yuriko Ito, Junya Nakade, Akihiro Seki, Ryosuke Gabata, Mitsuyoshi Okazaki, Shinichi Nakanuma, Arimi Fujita, Tsutomu Shimada, Taro Yamashita, Shintaro Yagi, Takumi Taniguchi, Yoshimichi Sai

**Affiliations:** 1 Department of Hospital Pharmacy, University Hospital, Kanazawa University, 13-1 Takara-machi, Kanazawa, Ishikawa 920-8641 Japan; 2 Department of Infection Control and Prevention, University Hospital, Kanazawa University, 13-1 Takara-machi, Kanazawa, Ishikawa 920-8641 Japan; 3 Department of Gastroenterology, Graduate School of Medicine, Kanazawa University, 13-1 Takara-machi, Kanazawa, Ishikawa 920-8641 Japan; 4 Department of Hepato-Biliary-Pancreatic Surgery and Transplantation, Graduate School of Medical Science, Kanazawa University, 13-1 Takara-machi, Kanazawa, Ishikawa 920-8641 Japan; 5 Department of Clinical Pharmacokinetics, Graduate School of Medical Sciences, Kanazawa University, 13-1 Takara-machi, Kanazawa, Ishikawa 920-8641 Japan; 6 Intensive Care Unit, Kanazawa University Hospital, 13-1 Takara-machi, Kanazawa, Ishikawa 920-8641 Japan; 7 AI Hospital/Macro Signal Dynamics Research and Development Center, Kanazawa University, 13-1 Takara-machi, Kanazawa, Ishikawa 920-8641 Japan

**Keywords:** High flow continuous hemodiafiltration, Anuric, Therapeutic drug monitoring, Vancomycin

## Abstract

**Background:**

High-flow continuous hemodiafiltration (HF-CHDF) combines diffusive and convective solute removal and is employed for artificial liver adjuvant therapy. However, there is no report on dosage planning of vancomycin (VCM) in patients with acute liver failure under HF-CHDF.

**Case presentation:**

A 20-year-old woman (154 cm tall, weighing 50 kg) was transferred to the intensive care unit (ICU) with acute liver failure associated with autoimmune liver disease. On the following day, HF-CHDF was started due to elevated plasma ammonia concentration. On ICU day 8, VCM was started for suspected pneumonia and meningitis (30 mg/kg loading dose, then 20 mg/kg every 12 hrs). However, on ICU day 10, VCM blood concentration was under the limit of detection (< 3.0 μg/mL) and the patient developed anuria. The VCM dose was increased to 20 mg/kg every 6 hrs. Calculation with a one-compartment model using the HF-CHDF blood flow rate as a surrogate for VCM clearance, together with hematocrit and protein binding ratio, predicted a trough VCM blood concentration of 15 μg/mL. The observed concentration was about 12 μg/mL. The difference may represent non-HF-CHDF clearance. Finally, living donor liver transplantation was performed.

**Conclusion:**

We report an acute liver failure patient with anuria under HF-CHDF in whom VCM administration failed to produce an effective blood concentration, likely due to HF-CHDF-enhanced clearance. VCM dosage adjustment proved successful, and was confirmed by calculation using a one-compartment model.

**Supplementary Information:**

The online version contains supplementary material available at 10.1186/s40780-023-00283-0.

## Background

High-flow continuous hemodiafiltration (HF-CHDF) is used as artificial liver adjuvant therapy for blood purification in acute liver failure [1–3], since it efficiently removes small-molecular compounds such as ammonia (NH_3_) and pathogenic cytokines, and promotes emergence from hepatic coma [1, 3]. However, coadministered therapeutic drugs with low molecular weight and low protein binding rate are also easily removed [4, 5], so it is necessary to ensure an appropriate administration dosage and schedule for patients receiving drug treatments.

Vancomycin (VCM) is a glycopeptide antibiotic with activity against methicillin-resistant *Staphylococcus aureus* (MRSA). To ensure efficacy and to avoid adverse effects of VCM, therapeutic drug monitoring is critically important [6]. Furthermore, since VCM is removed by hemodiafiltration [7], individual dosage design is essential [8–10].

CHDF, used for renal replacement therapy, is a continuous dialysis method that reduces the blood and diafiltrate flow rates (generally, blood flow rate: about 80 ~ 100 mL/min > dialysis flow rate + filtration flow rate: about 10 ~ 25 mL/min) compared with normal dialysis. However, we experienced a case of vancomycin administration under HF-CHDF, involving continuous high-flow on-line hemodiafiltration (on-line HDF) (blood flow rate: 200 mL/min < dialysis flow rate + filtration flow rate: 600 mL/min) for 24 hrs. Although VCM clearance during 4 hrs of on-line HDF has been examined [11], there is no report on the dosage design of VCM during HF-CHDF.

Here, we report a one-compartment model developed to aid dosage planning of VCM in an acute liver failure patient with anuria who was treated with VCM while receiving HF-CHDF. The results of VCM monitoring are also presented.

## Case presentation

**Fig. 1:**
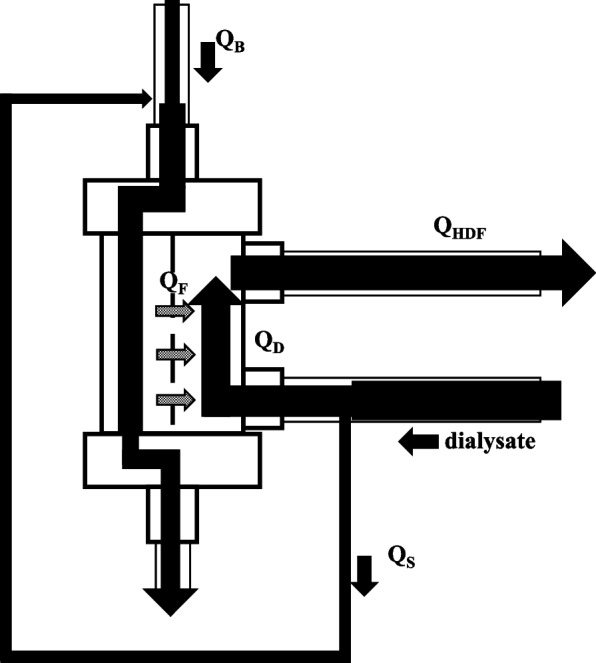
The HF-CHDF system used in this case. On-line hemodiafiltration in a predilution mode was performed continuously. Dialyzer: ABH-22PA (Asahi Kasei Medical Co., Ltd., Tokyo, Japan). Material: polysulfone membrane. Dialysate: Carbostar®・L (Yoshindo Inc., Toyama, Japan). Q_S_ = substitute fluid flow rate, Q_B_ = blood flow rate, Q_D_ = dialysis flow rate, Q_F_ = filtration flow rate, Q_HDF_ = dialysis outflow rate

A 20-year-old woman (154 cm tall, weighing 50 kg), who had been under long-term prednisolone treatment for dermatomyositis and autoimmune hepatitis, was hospitalized with acute liver damage. On the 11th day of hospitalization, her prothrombin time (PT) activity was 25% and NH_3_ level was 113 μg/mL. She was diagnosed with acute liver failure and transferred to the intensive care unit (ICU). Plasma exchange (PE) was conducted and steroid pulse therapy was started. On the following day (ICU day 2), HF-CHDF (Fig. 1: The system employs on-line HDF in a predilution mode) was started because the NH_3_ plasma concentration was elevated and she was diagnosed with coma II hepatic encephalopathy. Thereafter, HF-CHDF was mainly used in combination with PE and continuous plasma filtration with dialysis (CPDF; a combination of slow, continuous PE and hemodiafiltration [12]) to replenish coagulation factors and to control the NH_3_ level.

**Fig. 2:**
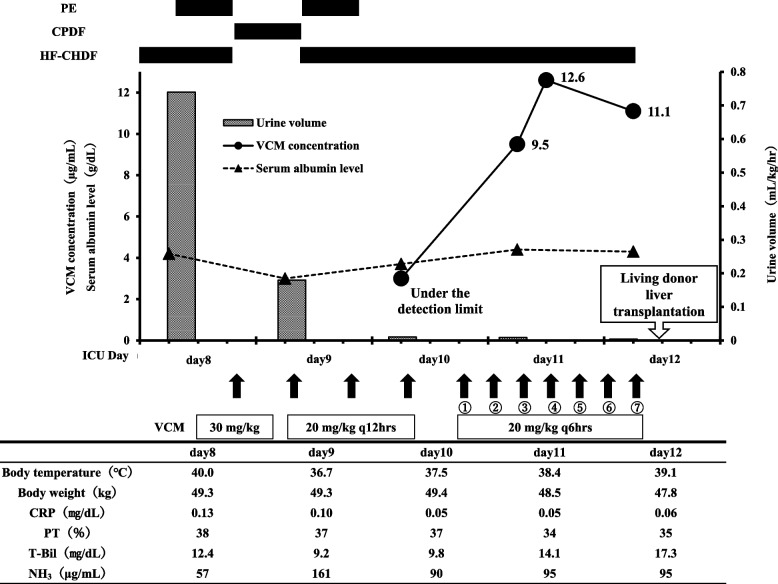
Clinical course and VCM concentrations from ICU day 8 to day 12. VCM: Vancomycin, PE: Plasma exchange, CPDF: Continuous plasma filtration with dialysis, HF-CHDF: High-flow continuous hemodiafiltration, CRP: C-reactive protein, PT: Prothrombin time, T-Bil: Total bilirubin, NH_3_: Ammonia

On ICU day 6, the patient developed fever at night, and piperacillin/tazobactam (PIPC/TAZ) treatment (4.5 g, every 6 hrs) was started for suspected ventilator-associated pneumonia (Culture result: Supplemental Table). On ICU day 7, cervical rigidity was observed, and PIPC/TAZ was changed to cefepime (CFPM) (2.0 g, every 12 hrs) for suspected meningitis. On ICU day 8, PE with HF-CHDF was changed to CPDF, and the antibiotic therapy was switched to VCM with meropenem (MEPM) (2.0 g, every 8 hrs) to achieve distribution to the cerebrospinal fluid (Fig. 2). At this time, accurate assessment of renal function was difficult because serum creatinine was removed by the dialysis, and the estimated glomerular filtration rate (eGFR) was more than 150 mL/min/1.73 m^2^. Since urine output was maintained (> 0.5 mL/kg/hr), and renal function before admittance to ICU was preserved (eGFR> 100 mL/min/1.73 m^2^), a loading dose of 30 mg/kg and maintenance dose of 20 mg/kg VCM every 12 hrs were adopted, according to the TDM Guidelines for Antibiotics 2016 [6] (Japan, The Japanese Society of Chemotherapy and the Japanese Society of Therapeutic Drug Monitoring).

On ICU day 9, due to elevated NH_3_ and prolonged disturbance of consciousness, dialysis was switched from CPDF to HF-CHDF again (flow rate: see Table 1), and PE was performed simultaneously for approximately 2 hrs. Meningitis was ruled out by spinal fluid examination, but fever and high inflammatory response persisted. VCM administration was continued because we could not rule out ventilator-associated pneumonia or catheter-related bloodstream infection associated with gram-positive cocci. On ICU day 10, the initial TDM for VCM was performed, and the blood level was under the detection limit (< 3.0 μg/mL), presumably due to the effects of HF-CHDF and PE. At the same time, the onset of anuria due to hepatorenal syndrome was noted. It was decided that a living donor liver transplantation (LDLT) would be performed 2 days later (ICU day 12), and HF-CHDF would be continuously performed until the day of LDLT. To maintain the VCM concentration in the therapeutic range prior to LDLT, the VCM dose was increased to 20 mg/kg every 6 hrs from the evening of ICU day 10 (MEPM was changed to 1.5 g every 6 hrs). The trough blood concentrations before the 3rd, and 4th (ICU day 11), and 7th (ICU day 12) VCM administrations were 9.5, 12.6, and 11.1 μg/mL, respectively. On ICU day 12, after the 7th administration of VCM, LDLT was conducted as scheduled and VCM administration was discontinued after the LDLT. No infection was apparent during VCM administration.

**Table 1:** HF-CHDF flow rate, VCM parameters, and patient factors

**HF-CHDF flow rate**	
Blood flow rate(Q_B_)	200 mL/min
Dialysis flow rate(Q_D_)	433 mL/min
Filtration flow rate(Q_F_)	167 mL/min
Substitute fluid flow rate (Q_S_)	167 mL/min
Q_HDF_ = Q_D_ + Q_F_	600 mL/min
**VCM parameters[**13, 14]	
Volume of distribution(Vd)	0.7 L/kg
Protein binding rate(fp)	0.35
Protein unbound form(fu)	0.65
**Patient factors**	
Body weight	50 kg
Hematocrit(Ht)	25%

## Discussion

In this case, administration of VCM according to the TDM guideline resulted in VCM concentrations below the therapeutic range during HF-CHDF. There is no report on the dosage design of VCM under conditions of enhanced clearance, such as HF-CHDF. We therefore adjusted the maintenance dosing schedule of VCM, and subsequent calculation of VCM blood concentration using a one-compartment model with the parameters shown in Table 1 supported the appropriateness of the adjusted dosage.

### Clearance calculation

The total body clearance (CL_tot_) under dialysis is expressed as follows,

1 $${\textrm{CL}}_{\textrm{tot}}={\textrm{CL}}_{\textrm{R}}+{\textrm{CL}}_{\textrm{NR}}+{\textrm{CL}}_{\textrm{HDF}}$$

CL_R_:renal clearance, CL_NR_:non-renal clearance, CL_HDF_:HF-CHDF clearance.

Since the patient was anuric on ICU day 10 and VCM is excreted almost exclusively from the kidneys, CL_tot_ could be approximated by CL_HDF_ (Eq. 2).

2 $${\textrm{CL}}_{\textrm{tot}}\approx {\textrm{CL}}_{\textrm{HDF}}$$

Clearance never exceeds the liquid inflow rate to dialyzer (Q_B in_) or the dialysis outflow rate (Q_HDF_ = Q_F_ + Q_D_), and the slower flow is rate-determining in blood purification therapy [4, 15–17]. If Q_B in_ < Q_D_ + Q_F_ and assuming complete removal of the drug from the blood, the maximum clearance is defined by Q_B in_.

3 $$\textrm{CL}={\textrm{Q}}_{\textrm{B}\ \textrm{in}}$$

Although Q_B in_ appears to be the blood flow rate (Q_B_) + substitute fluid flow rate (Q_S_) in the pre-dilution mode, the drug concentration is decreased by dilution before dialysis. Therefore, corrected clearance should be considered, since dilution reduce the efficacy of solute removal. In a previous report, corrected clearance (CL’) was defined [18] as follows:

4 $${\textrm{CL}}^{'}=\textrm{CL}\cdot {{\textrm{Q}}_{\textrm{BI}}}^{'}/{\textrm{Q}}_{\textrm{BI}}$$

where Q_BI_’ is the blood flow rate before dilution, i.e., Q_B_, and Q_BI_ is blood flow rate after dilution, i.e., Q_B_ + Q_S_. Substituting Eq. (3) into Eq. (4) gives the following result for CL’:

5 $${\textrm{CL}}^{'}={\textrm{Q}}_{\textrm{B}}$$

If the dialysis outflow rate (Q_HDF_ = Q_D_ + Q_F_) is greater than the blood flow rate (Q_B_), clearance is limited by Q_B_. In this case, Q_HDF_ was greater than Q_B_, so clearance is defined by Q_B_. In fact, only unbound drug in plasma is eliminated, so actual CL_HDF_ can be expressed as follows [4].

6 $${\textrm{CL}}_{\textrm{HDF}}={\textrm{Q}}_{\textrm{B}}\cdot \left(1-\textrm{Ht}/100\right)\cdot \textrm{fu}\cdot 60/1000\ \left(\textrm{L}/\textrm{hr}\right)$$

Substitution of Q_B_ = 200 mL/min under this condition, Ht = 25% in this patient and fu = 0.65 from the literature value of VCM (Table 1) gave a calculated CL_HDF_ value of 5.9 L/hr. The rate of disappearance (k_e_) of VCM in this patient was calculated as 0.17 hr^− 1^ based on Vd = 0.70 L/kg from the literature (Table 1) according to the following equation.

7 $${\textrm{k}}_{\textrm{e}}={\textrm{CL}}_{\textrm{tot}}/\textrm{Vd}\approx {\textrm{CL}}_{\textrm{HDF}}/\textrm{Vd}\ \left(/\textrm{hr}\right)$$

The t_1/2_ was calculated as 4.1 hrs using the formula t_1/2_ = ln2/k_e_.

### Calculation of blood concentration

Since VCM is homogeneously distributed under steady-state conditions, a one-compartment model was applied. The steady-state blood concentration of the drug during intermittent infusion was approximated as follows,

8 $${\textrm{R}}_0=\textrm{D}/{\textrm{t}}_0$$

9 $${\textrm{C}}_{\textrm{ss},\max }=\frac{\textrm{R}0}{{\textrm{k}}_{\textrm{e}}\cdot \textrm{Vd}\ }\ \left(1-{\textrm{e}}^{-\textrm{ke}\cdot \textrm{t}_0}\right)\ \left(\frac{1}{1-{\textrm{e}}^{-\textrm{ke}\cdot \uptau}}\right)\ \left(\upmu \textrm{g}/\textrm{mL}\right)$$

10 $${\textrm{C}}_{\textrm{ss},\min }={\textrm{C}}_{\textrm{ss},\max}\cdot {\textrm{e}}^{-\textrm{ke}\cdot \uptau}\ \left(\upmu \textrm{g}/\textrm{mL}\right)$$

R_0_: dosing rate, D: dose, t_0_: infusion time, τ: dosing interval, C_ss,max_: maximum blood concentration, C_ss,min_: minimum blood concentration.

To achieve a sufficiently high trough concentration while avoiding adverse effects, it is necessary to increase the frequency of dosing rather than the dosage amount, considering the short half-life of VCM. When a dose of 20 mg/kg was administered every 6 hrs, the values of C_ss,max_ and C_ss,min_ were calculated as 41.6 μg/mL and 15.2 μg/mL, respectively, from eq. 9 and 10. These values are suitable for VCM treatment, and based on the half-life of 4.1 hr, a steady state would be reached after the 3rd to 4th administration.

### Comparison of measured and calculated values

The measured blood concentration was around 12 μg/mL at the 4th (ICU day 11) and 7th (ICU day 12) VCM administrations after dose escalation to 20 mg/kg every 6 hrs at ICU day 10. This lies within the effective range for preventing infection before LDLT.

Nevertheless, this measured concentration (around 12 μg/mL) was 20% lower than the calculated steady-state concentration of around 15 μg/mL, presumably due to factors such as non-renal clearance and changes in protein binding rate.

The systemic clearance of VCM in healthy adults is about 100 mL/min, and the urinary excretion rate of unchanged drug is more than 90% [13]. However, VCM is slowly excreted via an unknown route in patients without renal function [19]. Indeed, non-renal clearance of vancomycin was suggested to amount to 1.05 L/hr/70 kg [20, 21]. Moreover, the protein binding rate influences VCM clearance [22]. We applied a protein binding rate of 0.35 based on literature values in our clearance calculation, but it remains possible that the protein binding fraction was different because of inadequate albumin synthesis due to liver failure [23], hyperbilirubinemia, and the effect of pre-dilution of blood flowing into the dialyzer [24], which may have increased the measured CL_HDF_. All these factors might have contributed to a blood concentration lower than the calculated value. Thus, there is scope to increase the accuracy of the calculation of blood concentration by taking account of these factors.

In recent years, several academic societies in America (e.g., the Infectious Diseases Society of America) have recommended the use of AUC/MIC as an important biomarker for efficacy and safety evaluation [25], not just the trough concentration of VCM. In 2022, the Japanese TDM guideline was similarly revised, so the calculation of AUC should be employed in future work.

## Conclusion

We report an acute liver failure patient with anuria under HF-CHDF who was treated with VCM. Dosage adjustment was required, and success was confirmed by calculation of VCM blood concentration using a one-compartment model. This calculation employs the HF-CHDF flow rate as a surrogate for clearance. Nevertheless, the measured VCM concentration was 20% lower than the calculated value (15 μg/mL), suggesting that other factors, such as non-renal clearance and protein binding rate, will need to be taken into account to improve the prediction of VCM concentration in patients under HF-CHDF.

## Supplementary Information

**Additional file 1.﻿**

## Data Availability

Not applicable.

## Abbreviations

CFPM: Cefepime
CL’: Corrected clearance
CL_tot_: Total body clearance
CL_NR_: Non-renal clearance
CL_R_: Renal clearance
CPDF: Continuous plasma filtration with dialysis
CRP: C-reactive protein
C_ss,max_: Maximum blood concentration
C_ss,min_: Minimum blood concentration
D: Dose
eGFR: Estimated glomerular filtration rate
fp: Protein binding rate
fu: Protein unbound form
HF-CHDF: High-flow continuous hemodiafiltration
Ht: Hematocrit
ICU: Intensive care unit
k_e_: Rate of disappearance
LDLT: Living donor liver transplantation
MEPM: Meropenem
MRSA: Methicillin-resistant *Staphylococcus aureus*
NH_3_: Ammonia
on-line HDF: On-line hemodiafiltration
PE: Plasma exchange
PIPC/TAZ: Piperacillin/tazobactam
PT: prothrombin time
Q_B_: Blood flow rate
Q_BI_: Blood flow rate after dilution
Q_BI_’: Blood flow rate before dilution
Q_B in_: Liquid inflow rate to dialyzer
Q_D_: Dialysis flow rate
Q_F_: Filtration flow rate
Q_HDF_: Dialysis outflow rate
Q_S_: Substitute fluid flow rate
R_0_: Dosing rate
t_0_: Infusion time
τ: Dosing interval
T-Bil: Total bilirubin
VCM: Vancomycin
Vd: Volume of distribution
