# Supplementary material for: Case report: therapeutic monitoring of vancomycin in an acute liver failure patient with anuria under high-flow continuous hemodiafiltration
Source: J Pharm Health Care Sci. 2023 May 1;9:15. doi: 10.1186/s40780-023-00283-0 (PMC10150540; doi:10.1186/s40780-023-00283-0)
Supplement: Supplementary file 1 — Additional file 1.﻿ [file 40780_2023_283_MOESM1_ESM.pptx]

## Slide 1
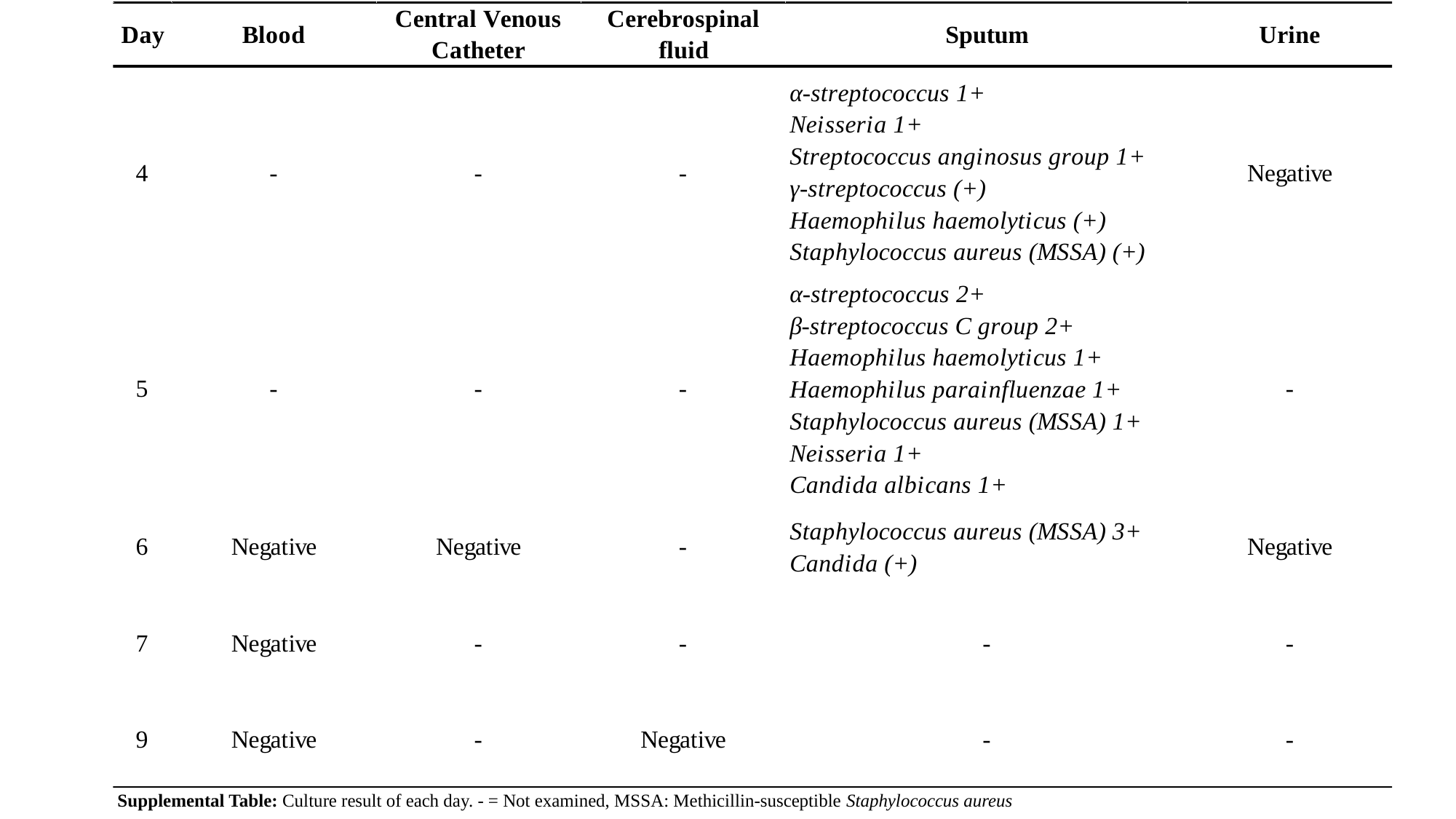

Supplemental Table: Culture result of each day. - = Not examined, MSSA: Methicillin-susceptible Staphylococcus aureus
